# Editorial: Towards Emotion AI to next generation healthcare and education

**DOI:** 10.3389/fpsyg.2024.1533053

**Published:** 2024-12-19

**Authors:** Yang Liu, Janne Kauttonen, Bowen Zhao, Xiaobai Li, Wei Peng

**Affiliations:** ^1^Center for Machine Vision and Signal Analysis, University of Oulu, Oulu, Finland; ^2^RDI & Competences, Haaga-Helia University of Applied Sciences, Helsinki, Finland; ^3^Guangzhou Institute of Technology, Xidian University, Guangzhou, China; ^4^School of Cyber Science and Technology, Zhejing University, Hangzhou, China; ^5^Department of Psychiatry and Behavioral Sciences, Stanford University, Stanford, CA, United States

**Keywords:** Emotion AI, education technologies, healthcare technologies, artificial intelligence, affective computing

Emotion AI, also known as affective computing or artificial emotional intelligence, has emerged as a transformative technology with significant potential to improve healthcare and education (Li et al., 2024a,b; Sun and Li, 2024). This field focuses on developing technologies capable of measuring, interpreting, simulating, and responding to human emotions, leveraging diverse data sources, including facial expressions, body gestures, vocal tones, and physiological and neural signals (Khare et al., 2024). Recent advancements in deep learning have significantly enhanced Emotion AI, but also bring critical challenges and concerns about data accessibility, reproducibility, user acceptance, and trust in the reliability of such systems. Emotion AI can enhance healthcare and education by monitoring and analyzing emotional states during activities. For example, educators can assess the cognitive and emotional aspects of learning, thereby providing a more holistic understanding of student engagement (Vedernikov et al., 2024). Personalized treatments and education adapt content to emotional responses, identifying struggles and offering tailored support. Clinicians benefit from objective progress tracking, automation of administrative tasks, and feedback on evidence-based practices.

On the technical side, both traditional, shallow feature-based methods (e.g., Support Vector Machines and Random Forest) and deep neural network models (e.g., LSTM and CNN types) have been utilized (Khare et al., 2024; Pepa et al., 2023). A key focus has been the development of multimodal systems that integrate diverse data sources, such as facial expressions, voice tone, dialogue sentiment, and physiological signals (Geetha et al., 2024). Multiple signals enable the aggregation of complementary information, leveraging the strengths of various measurement techniques to enhance robustness and accuracy.

Technology is only effective with users; thus, beyond developing methods, fostering adoption among professionals and citizens is essential. Alongside technical advancements, researchers have explored psychological and social factors influencing the acceptance and trust of AI systems, particularly in healthcare and education. Trust has proven pivotal for AI adoption, with studies investigating how to build it and what system attributes ensure trustworthiness (Li et al., 2024a,b). Key trust factors include explainability, transparency in processes and data usage, and the credibility of the institutions behind AI development.

This Research Topic explores novel theories, methodologies, and applications of Emotion AI in healthcare and education. The published works address technical aspects, models, and AI acceptance and literacy.

Burgess et al. evaluated automated facial coding software for parent-infant interactions across five studies with fathers and mothers. Automated detection rates were low (~25%) compared to manual coding in naturalistic settings but strongly correlated with manual assessments, particularly for positive expressions, when successful. Key challenges included poor lighting, facial occlusion, and rapid movements, highlighting the need for greater robustness in real-world conditions. Despite these limitations, the study demonstrated the potential of automated systems for analyzing authentic emotional expressions in parent-child interactions.

Başaran et al. introduced an innovative semi-supervised learning approach for stress monitoring. Using physiological data from 14 participants across five experiments, they achieved 77% accuracy with label propagation and 76% with deep autoencoders despite utilizing only 17% labeled data. Their method matched the performance of fully supervised approaches while substantially reducing annotation requirements, offering a practical solution for continuous stress monitoring in real-world healthcare applications.

Zhang and Cui introduced novel self-supervised learning methods for emotion recognition using EEG signals. Their experiments on SEED, SEED-IV, and DEAP datasets demonstrated effective feature representation learning without manual labels. Comparing three pre-training tasks—Relative Position, Temporal Shuffling, and Contrastive Predictive Coding (CPC)—they found CPC yielded the best results. This work addresses the challenge of limited labeled physiological data while achieving competitive performance.

Žvanut and Mihelič identified four distinct attitudes among older adults toward domestic social robots: Cautious Optimists, Skeptical Traditionalists, Positive Optimists, and Technophiles. Through interviews with 24 participants, they highlighted the influence of factors like technology familiarity, privacy concerns, and perceived utility on AI acceptance. Their findings offer valuable insights for designing emotionally intelligent robotic assistants tailored to diverse user needs and concerns.

Shen and Cui investigated the link between psychological needs satisfaction and AI literacy in 445 university students. Their findings revealed that technical and teacher support positively impacted students' autonomy and competence, subsequently improving AI literacy. Notably, satisfying psychological needs proved more critical than direct support in enhancing AI literacy. These insights offer valuable guidance for designing AI-enhanced educational environments that foster better learning and engagement.

Gong et al. examined patients' trust in AI-powered pharmacy intravenous admixture services across five studies. They found that patients generally trusted AI PIVAS less than human services, primarily due to a limited subjective understanding of AI systems. However, informed consent significantly improved trust by enhancing patients' understanding. This study underscores the critical role of transparent communication and psychological factors in adopting AI in healthcare.

The articles in this Research Topic advance methods for analyzing real-world datasets and explore how Emotion AI can be effectively integrated into healthcare and education, addressing challenges related to trust, privacy, and user acceptance. While Emotion AI shows promise, its use in these fields raises technical and ethical concerns, including data privacy, surveillance, and potential misuse of personal information. Clinical applications are further constrained by small sample sizes, lack of control groups, limited real-world testing, and methodological variability, which hinder reproducibility and reliability (Pepa et al., 2023). These studies tackle these challenges by developing novel model-training approaches for limited and noisy labeled data and identifying factors influencing AI acceptance and trust.

Future research should prioritize enhancing the robustness of emotion recognition in naturalistic settings, developing efficient learning methods to reduce dependence on labeled data, and addressing ethical considerations to support user acceptance of AI. Emphasis on cross-cultural validation and long-term evaluation in real-world applications is crucial. Recent generative AI (GenAI) foundation models, such as GPT-4, offer significant potential for advancing Emotion AI due to their versatility (Cheng et al., 2023). GenAI has already shown promise in recognizing emotional indicators (Elyoseph et al., 2024) and could help alleviate data scarcity challenges that hinder specialized model development. Additionally, there is growing interest in the emotional analysis of groups and crowds, broadening the field's scope (Veltmeijer et al., 2023; Li et al., 2024a,b).
